# Induction of anti-leukemic responses by stimulation of leukemic CD3+ cells with allogeneic stimulator cells

**DOI:** 10.1186/s40164-018-0118-5

**Published:** 2018-10-04

**Authors:** Alejandro Pando, John L. Reagan, Martha Nevola, Loren D. Fast

**Affiliations:** Division of Hematology/Oncology, Rhode Island Hospital and the Warren Alpert School of Medicine at Brown University, One Hoppin Street, Coro West Suite 5.0.1, Providence, RI 02903 USA

**Keywords:** Leukemia, Alloreactivity, T cells, Cytolytic T lymphocytes, Immunotherapy, Cross-reactivity

## Abstract

**Background:**

Immunotherapeutic protocols have focused on identification of stimuli that induce effective anti-leukemic immune responses. One potent immune stimulus is the encounter with allogeneic cells. Our group previously showed that the infusion of haploidentical donor white blood cells (1–2 × 10^8^ CD3+ cells/kg) into patients with refractory hematological malignancies induced responses of varying magnitude in over half of the patients. Because donor cells were eliminated within 2 weeks in these patients, it is presumed that the responses of recipient lymphocytes were critically important in achieving prolonged anti-leukemic responses.

**Methods:**

The role of patient CD3+ cells in anti-leukemic responses was examined by isolating peripheral blood mononuclear cells from newly diagnosed leukemic patients. Immunophenotyping was performed on these peripheral blood mononuclear cells. CD3+ cells were isolated from the peripheral blood mononuclear cells and tested for their ability to proliferate and lyse autologous leukemic cells when stimulated with unrelated allogeneic cells.

**Results:**

Allostimulated CD3+ cells effectively generated cytolytic responses to autologous CD3-cells in 11/21 patients. Increased numbers of CD4+ cells expressing high levels of granzyme A, B and perforin and CD8+CD39+ cells were found in nonresponsive CD3+ cells.

**Conclusions:**

These results indicate that CD3+ cells from leukemic patients are capable of generating anti-leukemic responses when stimulated with unrelated allogeneic cells. This model can be used to identify approaches using alloreactive responses by patient lymphocytes to enhance in vivo anti-leukemic responses.

## Background

Allogeneic cells are one of the more potent stimuli of the immune system, as 100–1000 fold more cells respond to an allogeneic major histocompatibility complex (MHC) molecules than to a foreign peptide presented by self MHC molecules [1]. Alloreactivity is the driving force for deleterious responses such as graft-versus-host disease (GVHD) and organ rejection seen in transplantation, but is also linked to beneficial graft versus leukemia (GVL) responses which has led to efforts to separate GVL responses from GVHD responses [2]. Within our group a nonengraftment donor leukocyte infusion protocol, in which infusion of large numbers of haploidentical cells at a dose of 1–2 × 10^8^ CD3+ cells/kg into patients with refractory hematological malignancies, resulted in responses of varying magnitude in over half of the patients tested (14/26 overall responses with 5 complete responses) including three complete responses in patients with refractory acute myeloid leukemia (AML) [3]. These responses occurred in the absence of durable engraftment as donor cells were no longer detected within 2 weeks of infusion. This protocol was characterized by the rapid development of a cytokine release syndrome (CRS) marked by high plasma levels of proinflammatory cytokines and fevers that remitted with corticosteroid administration. Anti-leukemic responses may potentially be mediated by donor lymphocytes for a limited period of time but, given the lack of durable chimerism, we postulate that extended responses were more likely mediated by recipient lymphocytes [4]. The intense alloreactive immune response generated by this protocol could facilitate anti-leukemic responses by a variety of mechanisms, such as the reactivation of memory anti-T cells from leukemic patients [5, 6] or the cross reactivity of patient alloreactive effector cells toward patient cancer cells [1, 7]. Herein we describe the ability of leukemic patient CD3+ lymphocytes to generate cytolytic responses against the syngeneic CD3-fraction, which includes leukemia cells, in vitro following stimulation with allogeneic donor peripheral blood mononuclear cells (PBMC).

## Methods

### Patient samples

The patient peripheral blood mononuclear (PBMC) samples included newly diagnosed acute lymphoid, acute myeloid, chronic myeloid, and chronic myelomonocytic leukemic patients categorized based on WHO criteria. Stimulator PBMCs were obtained from unrelated normal volunteers who were employees at Rhode Island Hospital. HLA typing was not performed on patient or donor samples. Patient and donor samples were collected following informed consent through a Rhode Island Hospital IRB approved protocol.

After isolation of PBMC using Ficoll-Hypaque discontinuous centrifugation, CD3+ and CD3-cells were isolated from the patient’s PBMC using CD3 immunomagnetic particles according to manufacturer’s instructions (Miltenyi Biotec, Inc, San Diego, CA, USA). Aliquots of the CD3-fraction of the patient’s cells, containing the leukemic cells, were frozen for later use. Blood from two unrelated normal donors were obtained for each patient. The PBMC obtained from the blood of each normal control along with the remainder of the CD3-cells from the leukemic patient were treated with Mitomycin C (Sigma, St. Louis, MO, USA).

### Immunophenotyping

Immunophenotyping was conducted on the freshly isolated PBMC from the leukemic patients. The CD3+ and CD3-cells were stained with antibody panels which defined T cell subsets and NK cells (APC-H7 anti-CD3, V500 anti-CD4, V450 anti-CD8, FITC anti-TCRγδ, PerCP-Cy5.5 anti-CD56), differentiation and activation (FITC anti-CD27, PE anti-CD45RA, FITC anti-CD25, APC anti-CD39, PerCP-Cy5.5 FoxP3, APC anti-CD152, FITC anti-CD279, PE anti-Tim3,) and cytolytic activity (FITC anti-granzyme A, Alexa 647 anti-granzyme B, PE anti-perforin, FITC anti-CD107a, PE anti-TRAIL, Alexa 647 anti-Fas ligand). All antibodies were purchased from BDBiosciences (San Jose, CA).

### Assays

Mixed lymphocyte cultures (MLC) were set up using RPMI medium containing 5% Human AB serum (Valley Biomedical, Winchester, VA, USA) and 1% penicillin and streptomycin (Life Technologies, Grand Island, NY, USA) [8]. 1 × 10^6^ patient CD3+ cells or 2 × 10^6^ control PBMC were added to 2 × 10^6^ mitomycin C treated PBMC from controls or CD3-leukemic fraction in 2 mL per well in a 24 well plate. In addition, triplicate wells containing tenfold fewer cells/medium were prepared in a 96 well plate. The response of T cells from leukemic patient samples to bound anti-CD3/CD28 in a prepared 96 well plate was tested by plating 1 × 10^5^ T cells from leukemic patients/well or 2 × 10^5^ PBMC (normal controls)/well, each in triplicate. All plates were incubated at 37 °C under 10% CO_2_ for the designated period of time.

Proliferation of cells was determined by measuring ^3^H-thymidine incorporation after a 4 h pulse on day 3 for cells activated with anti-CD3/28 or day 5 for MLC cultures. The data is presented as stimulation index (S.I.), equal to the ratio of the mean counts per minute (cpm) for the response to allogeneic stimulators divided by the sum of responder vs. medium and stimulator vs. medium cpm. Effector cells generated by 7 day MLC cultures were collected and tested for their ability to lyse ^51^Cr labeled target cells (both CD3-leukemic cells and normal donor blasts) using varying effector target cell ratios. Donor blasts were generated by culturing PBMC in 125U/mL rhuIL-2 (Zeptometrix, Buffalo, NY, USA) and 2 ug/mL concanavalin A (Sigma, St. Louis, MO, USA) in RPMI 1640 containing 5% FCS and streptomycin/penicillin for 7 days at 37 °C. Leukemic CD3-cells were thawed on day 6 and held overnight in 20% FCS medium. The results are presented as lytic units/10^6^ cells in which 1 lytic unit (LU) is defined as the number of effector cells required to achieve 30% lysis of 1 × 10^4^ target cells.

### Statistical analysis

Statistical analysis was performed utilizing Graphpad Prism 7.0 (GraphPad Software). The Mann–Whitney non-parametric test (Mann–Whitney U = 248, n_1_ = n_2_ = 42, P < 0.05 two-tailed) and unpaired t-tests were used to determine the P values indicated in the figures. Significance was defined to have been reached at P values from < 0.05.

## Results

The characteristics of 21 newly diagnosed leukemic patients studied are described in Table 1. Cytogenetic and molecular classifications were based on NCCN criteria [9]. The median age was 60.5 years (range, 29–86), the median number of absolute lymphocyte count collected was 2.7 × 10^6^/mL, and the median percentage of peripheral blasts in the CD3-cells was 23%. Disease types were (AML, 14 patients), chronic myeloid leukemia (CML, 1 patient), chronic myelomonocytic leukemia (CMML, 3 patients), and acute lymphocytic leukemia (ALL, 3 patients).

**Table 1:** Characteristics of newly diagnosed leukemic patients

Pt#	Sex	Age	Diagnosis	WBC	ALC	Peripheral blasts (%)	Cytogenetics molecular markers
1	Female	74	AML	17.1	6.2	6	XX
2	Female	65	AML	7.4	3.6	9	t (4;11), Tetrasomy 8
3	Male	26	CML	336.5	20.2	4	t (9;22)
4	Male	31	AML	9.2	2.7	39	inv (3), Monosomy 7
5	Female	86	AML	54.4	8.2	32	Trisomy 13
6	Male	32	AML	7.1	0.9	12	del (17), t (16;21)
7	Male	84	CMML	39.6	2.4	11	XY
8	Male	62	AML	19.4	2.5	23	inv (16), del (7)
9	Female	29	AML	8.6	4	47	t (11;20), FLT3 ITD+
10	Male	64	AML	138.3	16.6	80	XY, FLT3 ITD+
11	Female	70	AML	12.8	2.4	33	t (11;19)
12	Male	56	CMML	14.4	2.2	14	XY
13	Female	48	CMML	16.3	2.6	19	XX
14	Male	59	ALL	5.1	2.7	45	t (9;22)
15	Female	34	ALL	341.8	23.9	88	XX
16	Male	46	AML	18.5	2.3	73	t (15;17)
17	Male	64	AML	1.2	0.2	3	XY, FLT3 ITD+
18	Female	57	AML	34.5	1.7	9	XX inv (16)
19	Female	32	ALL	58.1	7.3	86	XX Ph+
20	Male	62	AML	28.1	8	9	XY
21	Female	77	AML	84.4	4.2	81	Not sent

**Fig. 1:**
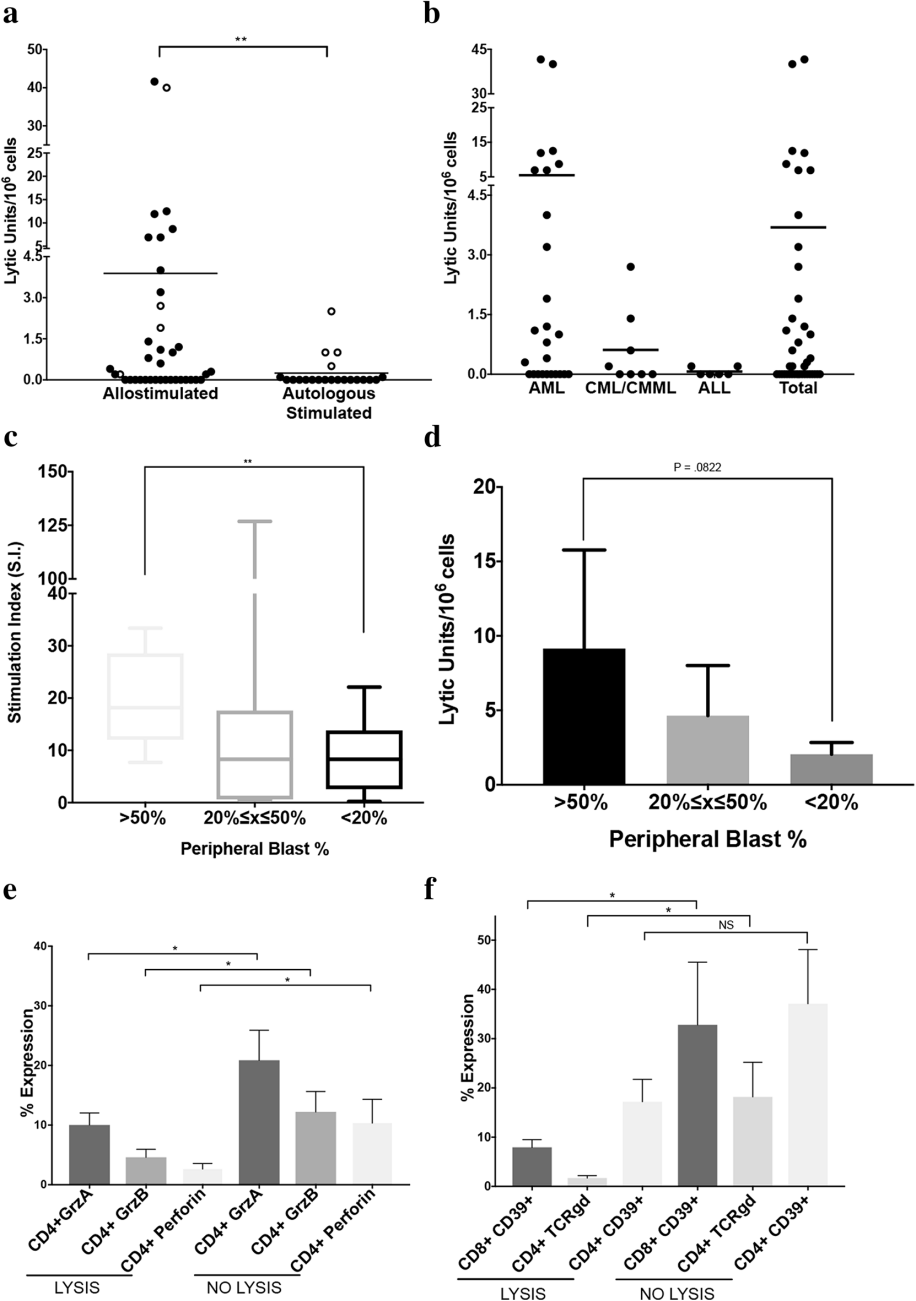
**a** Lysis (lytic units/10^6^ cells) of CD3-cells observed in CD3+ cells incubated with mitomycin-treated healthy donor allogeneic PBMC (allostimulated CD3+ cells) or syngeneic CD3-cells (autologous stimulated CD3+ cells). Open circles represent the 4 patient samples that demonstrated spontaneous ability to lyse CD3-cells and their corresponding ability to lyse once allostimulated. (Mann–Whitney U = 248, n_1_ = n_2_ = 42, P < 0.05 two-tailed). **b** Lysis (lytic units/10^6^ cells) of CD3-cells observed by diagnosis. **c** Stimulation index of allostimulated CD3+ cells separated into greater than 50% peripheral blasts, between 20 and 50% peripheral blasts, or less than 20% peripheral blasts. **d** Lysis (lytic units/10^6^ cells) of CD3-cells observed in allostimulated CD3+ cells separated into greater than 50% peripheral blasts, between 20 and 50% peripheral blasts, or less than 20% peripheral blasts. **e** Percentage of CD4+ cells expressing Granzyme A, CD4+ Granzyme B, and CD4+ Perforin on the leukemic patient CD3+ population that demonstrated lysis and the population that did not lyse (mean ± standard deviation (s.d.)). **f** Percentage of cells expressing CD8+CD39+, CD4+ TCRγδ, and CD4+CD39+ on the leukemic patient CD3+ population that demonstrated lysis and the population that did not lyse (mean ± S.D.). *P < 0.05. *NS* not significant. Horizontal bars in (**a**) and (**b**) signify mean

CD3+ cells were either incubated with mitomycin C-treated healthy unrelated donor allogeneic PBMC (allostimulated CD3+ cells) or syngeneic CD3-cells (autologous stimulated CD3+ cells). Proliferation was detected for all combinations with similar levels of proliferation between those cells stimulated with allogeneic cells (S.I. 13.2 ± 19.3) or autologous CD3-cells (S.I. 15.3 ± 21.1). On day 7, allostimulated CD3+ cells and CD3+ cells stimulated with syngeneic CD3-cells were examined for cytolytic killing of CD3-cells. Eleven of the twenty-one patient allostimulated CD3+ cells were able to lyse CD3-cells (Fig. 1a). CD3+ cells from seven of the responding leukemic patients lysed CD3-cells when stimulated with either allogeneic stimulator. CD3+ cells from four patients were able to lyse CD3-target cells when stimulated with syngeneic CD3-cells. These CD3+ cells also proliferated strongly to the syngeneic CD3-cells (S.I. of 3.9, 20.9, 24.2, 79.6) and three out of four also generated strong alloreactive CTL when stimulated with allogeneic cells. These findings contrasted to the lack of proliferation seen when CD3+ cells obtained from normal donors were stimulated with syngeneic CD3-cells (SI = 0.9 ± 0.3, n = 4). In addition no detectable lysis of normal syngeneic CD3-cells was seen when CD3+ cells from normal donors were stimulated with allogeneic PBMNC (n = 9), syngeneic PBMNC (n = 4) or syngeneic CD3-cells (n = 4).

T CD3-cell killing was induced via allogeneic stimulation for AML and CML/CMML but not in ALL samples (Fig. 1b). Patient samples with peripheral blasts greater than 50% had an increase in the stimulation index compared to those with less than 20% peripheral blasts. Additionally, lytic effector function in greater then 50% blasts had a P value of 0.08 compared to those with less than 20%. Together this suggests that candidates with more peripheral blasts may respond better to allostimulation than those with less peripheral blasts.

Analysis of factors that were associated with the ability or inability of the alloreactive CD3+ cells to generate cytolytic activity toward syngeneic CD3-cells containing leukemic cells identified several immunophenotypes associated with the CD3+ cells which did not generate cytolytic activity toward the CD3-cells. The first was an increased number of CD4+ cells expressing high levels of perforin, granzyme A and granzyme B (Fig. 1c). A second finding was an increased number of CD8+ cells expressing CD39, a phenotype found in terminally exhausted CD8+ cells. A nonsignificant increase of CD4+CD39+cells was also observed in this set of CD3+ cells. (Figure 1d). In addition the number of CD4+ cells expressing gamma delta T cell receptor (TCRγδ) was significantly upregulated on in the unresponsive CD3+ cells (Fig. 1d). No significant differences in the number of FoxP3+ cells or the expression of inhibitory receptors was detected between the patients that lysed leukemic cells and those that did not. Staining freshly isolated cells with antibodies detecting cytolytic activity or pathways including CD107a, TRAIL and Fas Ligand did not demonstrate any predictive value for identifying which CD3+ cells would generate anti-leukemic responses (data not shown). Interestingly, all three AML patients who harbored FLT-3 internal tandem duplication (ITD) mutations, a prognostic marker in AML with poor prognosis [10], were able to lyse leukemia containing CD3-cell fractions following allogeneic stimulation.

## Discussion

In summary, in our screening of 21 newly diagnosed leukemia samples approximately half of the patients’ CD3+ cells were able to generate lysis of syngeneic cells containing leukemic cells after allostimulation. The ability of alloreactive effector cells to lyse syngeneic leukemic cells was similar to the findings previously reported in mice [11, 12]. It was even reported that stimulation with syngeneic tumor cells induced alloreactive effector cells [13]. These findings would suggest that cancer cells express unique peptides on their MHC molecules that allow them to be recognized by alloreactive effector cells [14]. Previously, when the peptides bound to HLA B7 molecules isolated from normal cells and leukemic cells were analyzed and compared, it was observed that there were many more unique peptides present on the HLA B7 molecules isolated from the leukemic cells [15]. Many of these unique peptides were phosphopeptides. Stimulation of PBMC from normal donors with a pool of these unique peptides resulted in T cell responses while the same pool of peptides did not induce responses in PBMC from cancer patients. This suggested that those effector cells had been inhibited or eliminated in the cancer bearing patients.

While one possibility is that alloreactive T cells could cross react with tumor cells, another possibility is that the alloreactive responses activate T cells specific for tumor associated antigens. For example, T cells reactive with Wilms tumor-1 (WT1) antigen have been identified in many individuals [16–20]. Guo et al. [17] found increased numbers of Wilm tumor-1 (WT1) antigen reactive CD8+ T cells in 33 out of 39 patients that received infusions of HLA-mismatched donor cells in their protocol. Although the source of these antigen reactive CD8+ cells was unknown in many of these cases, in 6 of these cases the WT1 reactive CD8+ cells could be determined to be of host origin. Several studies have also indicated that vaccination with WT1 vaccines facilitated or prolonged responses [21, 22]. A study in which patients received G-CSF mobilized haploidentical donor cells after receiving 100 cGy total body irradiation, a patient who went into complete remission was found to have high levels of CD8+ cells reactive with proteinase-3, a tumor associated antigen, with no detectable donor cells [23]. These examples indicate that the expansion of T cells reactive with antigens expressed by the leukemic cells could distinguish between those individuals whose cells responded in our study and those that did not.

Phenotypic studies identified an increased number of CD4+ cells expressing cytolytic effector molecules and increased number of recipient CD8+ cells expressing CD39, a marker of exhaustion as a characteristic of the CD3+ cells that did not generate anti-leukemic responses when stimulated with allogeneic cells. The appearance of cytolytic CD4+ cells can be linked to repeated antigen exposure in chronic infection and in cancer bearing individuals reflecting a senescent immune system [24]. It has been shown that these cells can mediate cytolytic activity either via the granzyme perforin pathway or via Fas/Fas ligand signaling. In patients with chronic hepatitis B infection and hepatocellular carcinoma it was found that the CD4+ CTL also produced IL-10 which inhibited the function of CD8+ CTL [25]. Adding anti-IL-10 antibody reversed the inhibition of CD8+ cell function mediated by the CD4+ CTL. This mechanism could provide a possible explanation for the association between increased numbers of CD4+ CTL and decreased CD8+ cell activity.

In our experiments, the increased numbers of CD4+ expressing the γδTCR was associated with lack of response. Usually γδ T cells have an advantage over αβ T cells in cancer because antigen processing and presentation is not required but can instead directly recognize cancer molecules [26] although some γδ subsets may facilitate cancer growth [27]. There is a recent report of CD39+ γδ T cells that were induced by TGF-β and served as potent immunoregulatory cells in colorectal cancer [28]. Although it cannot be determined directly from this data because antibodies to CD39 and γδ TCR were included in different antibody panels, increased numbers of both CD4+ γδ T cells and CD39+CD4+ cells were detected in the CD3+ cells from the unresponsive patients. These findings would be consistent with an increased number of these CD39+ γδ T regulatory cells in this group of patients that are inhibiting other T cell responses and this possibility will be examined more closely in future studies. The CD8+CD39+ phenotype is associated with the exhausted phenotype and increased numbers of these cells suggests that the cancer reactive CD8+ cells could be exhausted and unable to respond to allostimulators [29].

In future clinical trials of nonengraftment donor leukocyte infusion protocols, it will be interesting to test if the expression of these particular phenotypes will identify those patients unresponsive to this protocol. These in vivo trials will further define which factors allow for reproducible induction of an anti-leukemic response by allostimulated leukemic patient CD3+ cells.

## Conclusions

Patient’s T lymphocytes are frequently able to attack syngeneic hematological malignancies when stimulated with allogeneic cells. Nonresponders can be defined using the phenotypic distinctions based on the presence of CD4+ CTL and CD8+CD39+ cells. Understanding the basis for the ability of alloreactive cells to lyse syngeneic cancer cells will allow for the development of protocols which harness these responses to achieve effective anti-cancer responses in all patients.

## Abbreviations

CRS: cytokine release syndrome
CTL: cytolytic T lymphocyte
GVHD: graft-versus-host disease
GVL: graft-versus-leukemia
MHC: major histocompatibility complex
MLC: mixed lymphocyte culture
PBMC: peripheral blood mononuclear cells
